# Impact of Salinomycin on human cholangiocarcinoma: induction of apoptosis and impairment of tumor cell proliferation *in vitro*

**DOI:** 10.1186/1471-2407-12-466

**Published:** 2012-10-11

**Authors:** Thorsten Lieke, Wolf Ramackers, Sabine Bergmann, Jürgen Klempnauer, Michael Winkler, Johannes Klose

**Affiliations:** 1 Department of General, Visceral and Transplantation Surgery, Hannover Medical School, Hannover, Germany

**Keywords:** Salinomycin, Cholangiocarcinoma, Apoptosis, Tumor cell migration, Cell cycle

## Abstract

**Background:**

Cholangiocarcinoma (CC) is a primary liver cancer with increasing incidence worldwide. Despite all efforts made in past years, prognosis remains to be poor. At least in part, this might be explained by a pronounced resistance of CC cells to undergo apoptosis. Thus, new therapeutic strategies are imperatively required. In this study we investigated the effect of Salinomycin, a polyether ionophore antibiotic, on CC cells as an appropriate agent to treat CC. Salinomycin was quite recently identified to induce apoptosis in cancer stem cells and to overcome apoptosis-resistance in several leukemia-cells and other cancer cell lines of different origin.

**Methods:**

To delineate the effects of Salinomycin on CC, we established an *in vitro* cell culture model using three different human CC cell lines. After treatment apoptosis as well as migration and proliferation behavior was assessed and additional cell cycle analyses were performed by flowcytometry.

**Results:**

By demonstrating Annexin V and TUNEL positivity of human CC cells, we provide evidence that Salinomycin reveals the capacity to break apoptosis-resistance in CC cells. Furthermore, we are able to demonstrate that the non-apoptotic cell fraction is characterized by sustainable impaired migration and proliferation. Cell cycle analyses revealed G2-phase accumulation of human CC cells after treatment with Salinomycin. Even though apoptosis is induced in two of three cell lines of CC cells, one cell line remained unaffected in regard of apoptosis but revealed as the other CC cells decreased proliferation and migration.

**Conclusion:**

In this study, we are able to demonstrate that Salinomycin is an effective agent against previously resistant CC cells and might be a potential candidate for the treatment of CC in the future.

## Background

Cholangiocarcinoma (CC) is an adenocarcinoma arising from the biliary epithelial cells and can affect both the intra- and extrahepatic biliary tree [1]. Beside hepatocellular carcinoma (HCC) it is the most common liver cancer with increasing incidence over the past years [2-4]. While in Asian countries the high incidence of CC is associated with liver flukes also in Northern America and Europe intrahepatic CC occurs in increasing number of unknown reason [4]. Patient´s survival is dramatically restricted due to limited treatment options and advanced stage of disease at presentation [5]. Thus treatment of CC is currently one of the biggest challenges in modern oncology. The only curative treatment options for this kind of cancer are radical surgical resection or as performed in some centers for a selected subset of patients liver transplantation [5,6]. Chemotherapy is less effective; however, a new protocol combining Gemcitabine and Cisplatin might be a promising therapeutical strategy for patients with advanced CC [7]. Limited data is available on the exact pathomechanisms leading to the development of CC. Chronic inflammatory conditions, such as primary sclerosing cholangitis, congenital biliary disorders, infection with liver flukes or toxic agents are supposed to be related to the malignant transformation of the biliary epithelial cells [8].

Salinomycin is a polyether antibiotic, originally isolated from *Streptomyces albus*[9]. It acts as a potassium ionophore and thereby interferes with transmembrane potassium potential, leading to mitochondrial and cellular potassium efflux [10,11]. Salinomycin is widely-used as an anticoccidial in poultry [12] and as a dietary supplement in ruminants` and pigs` breed [13,14]. Recently, the potential of Salinomycin as an anti-cancer agent has been elucidated [15]. First, the effects of Salinomycin were described in the treatment of cancer stem cells *in vitro* and *in vivo*[16]. Later, the efficacy of Salinomycin against tumor cells has been demonstrated in several cancer cell lines from different origin, including solid and non-solid malignancies [17-20]. Nevertheless, the precise mode of action of Salinomycin as an anti-cancer agent remains unclear.

So far, the impact of Salinomycin treatment on human CC cells has not been investigated. Thus, the aim of the present study was to investigate whether the anti-cancer effect of Salinomycin is also sufficient for the treatment of CC. We identified that Salinomycin induces apoptosis in human CC cells *in vitro*. In addition, we demonstrate that Salinomycin impairs tumor cell migration, reduces tumor cell proliferation and leads to cell cycle accumulation. Our data provide that treatment of human CC cells with Salinomycin has a promising anti-cancer effect.

## Methods

### Cell lines and culture

For proof of principle of the properties of Salinomycin the reactivity of three well characterized human CC cell lines, Mz-ChA-1 , TFK-1 and EGI-1 [21-23] was tested. Cells were cultured at 37°C and 5% CO_2_ in culture medium (RPMI 1640 + Glutamax, supplemented with 10% fetal bovine serum, 10 mM HEPES-Buffer, 1% MEM non-essential Amino acids, penicillin (50 U/ml), and streptomycin (50 mg/l)) (Invitrogen, Darmstadt, Germany). Medium was changed every 48 hours. Mz-ChA-1 cells were a kind gift from Dr. A Knuth (Universitiy Hospital of Zurich, Zurich, Switzerland). TFK-1 and EGI-1 cells were provided by S. Zender (Hannover Medical School, Hannover, Germany). Jurkat cells were cultured in RPMI 1640, supplemented with 10% fetal bovine serum, penicillin (50 U/ml) and streptomycin (50 mg/l), at 37°C and 5% CO_2_. Cells were maintained by passages every 72 hours.

### Chemicals

Salinomycin was purchased from Sigma-Aldrich (Munich, Germany) and dissolved in DMSO. Gemcitabine was purchased from TEVA (Radebeul, Germany) and dissolved in phosphate buffered saline (PBS). Stock solutions were stored at −20°C.

### Proliferation assay

1 × 10^3^ human CC cells were cultured in medium alone or in the presence of 1 mM Gemcitabine, 1 μM, 2 μM, 5 μM or 10 μM Salinomycin in 96-well flat bottom plates. The cultures were expanded for different time periods: either 24 or 48 hours under treatment of reagents respectively or first exposed to Gemcitabine and Salinomycin for 48 hours followed by additional 48 hours in medium alone. For the last 16 hours of culture cells were pulsed with 1 μCi ^3^H-Thymidine and incorporation was assessed using a β-counter (LKB Wallac, Turku, Finland).

### Cell cycle analysis

Cell cycle analysis was performed using the CellTest Plus Reagent Kit (Becton Dickinson Imunocytometry Systems, San Jose, California, USA). 1 × 10^5^ human CC cells were seeded in 12-well plates for 24 hours without reagents to allow attachment. Cells were then incubated in the presence or absence of 1 mM Gemcitabine, 1 μM, 2 μM, 5 μM or 10 μM Salinomycin for 24 hours, trypsinized and stained according to the manufacturer´s instructions. Analysis was performed using a FACSCalibur (BD Bioscience, Heidelberg, Germany) and the ModFit LD software (Verity House Software, Topsham, Maine, USA).

### Migration assay

Tumor cell migration was analyzed using a transwell chamber (Corning Coster, Corning, NY, USA) provided with an 8 μm pore polycarbonate membrane. Human CC cells were placed at 5 × 10^5^ cells/well in culture medium containing 10% fetal calf serum in the upper compartment of the chamber. The lower compartment was filled with culture medium containing 30% fetal calf serum acting as a chemo-attractant [24]. Cells were cultured in the absence or presence of 1 mM Gemcitabine, 1 μM, 2 μM, 5 μM or 10 μM Salinomycin for 48 hours. The membrane was then removed, fixed with ethanol and stained with hematoxylin. The membranes were analyzed under a light microscope counting the number of migrated cells to the lower surface of the membrane in five randomly selected fields as described before [25].

### Annexin V analysis

Human CC cells were plated in 6-well plates at 1 × 10^6^ cells/well in culture medium and grown until confluence. Cells were further incubated in the presence or absence of 1 mM Gemcitabine, 1 μM, 2 μM, 5 μM or 10 μM Salinomycin for 24 hours. Cells were trypsinized and washed two times with PBS. Induction of apoptosis was assessed using Annexin V apoptosis detection kit (BD Biosciences, Heidelberg, Germany) according to the manufacturer`s instructions. Analysis was performed with a FACSCalibur (BD Biosciences).

### Terminal desoxynucleotidyl transferase (dUTP) nick end labeling (TUNEL) assay

3 × 10^4^ human CC cells were cultured in 8-well glass chamber slides (Nunc, Rochester, NY, USA) until confluence in medium alone and further on for 24 hours in the absence or presence of 1 mM Gemcitabine, 1 μM, 2 μM, 5 μM or 10 μM Salinomycin. Cells were fixed with 4% paraformaldehyde for 25 min at 4°, washed with PBS and permeabilized by methanol/acetone solution for 10 min. Cells were equilibrated with equilibration buffer (Promega, Madison, Wisconsin, USA) for 5–10 min at room temperature. After washing with PBS cells were incubated with rTDT incubation buffer for 60 min at 37°. Reaction was stopped with 20x concentrated SSC buffer and washed with PBS. Nuclei staining was performed by adding DAPI 1:2500 (Sigma, St. Louis, Missouri, USA) in PBS during the final washing procedure. Cells were mounted in VECTASHIELD and analyzed within 24 hours. TUNEL assay was performed using a commercial kit (Promega, Madison, Wisconsin, USA).

### Immunocytochemistry

3 × 10^4^ adherent human CC cells were cultured in chamber slides in medium until confluence. Cells were further cultured in the absence or presence of 1 mM Gemcitabine, 1 μM, 2 μM, 5 μM or 10 μM Salinomycin for 24 hours. Cells were fixed with 4% paraformaldehyde for 25 min at 4°, washed with PBS and air-dried for 1 hour. As positive control for Caspase-dependent induction of apoptosis human Jurkat cells were exposed to human TRAIL-expressing transgenic fibroblasts for 12 hours. Jurkat cells were subsequently washed from adherent fibroblasts and air-dried on slides after cytospin centrifugation (Shandon Cytospin GMI, Ramsay, Minnesota, USA). All samples were fixed by acetone/methanol solution and afterwards air-dried for one hour. Analysis of activated caspases was performed using a monoclonal antibody against cleaved caspase-3 (New England Biolabs, Ipswich, Massachusetts, USA) diluted 1:200 in PBS. Staining was visualized using Cy3-labeled secondary donkey anti-rabbit antibody (Biolegend, San Diego, California, USA) diluted 1:500 in PBS. Nuclei staining was performed by adding DAPI 1:2500 in PBS during the final washing procedure. Cells were analyzed using the AxiolmagerM1 microscope (Zeiss, Jena, Germany) and the AxioVision 4.6 software (Zeiss).

### Statistical analysis

Results were expressed as mean ± SD. All experiments were performed at least in three individual experiments. Results were analyzed for statistical significance using two-way ANOVA test or student´s t-test.

## Results

### Exposure of Salinomycin to human CC cells provokes morphological changes

**Figure 1:**
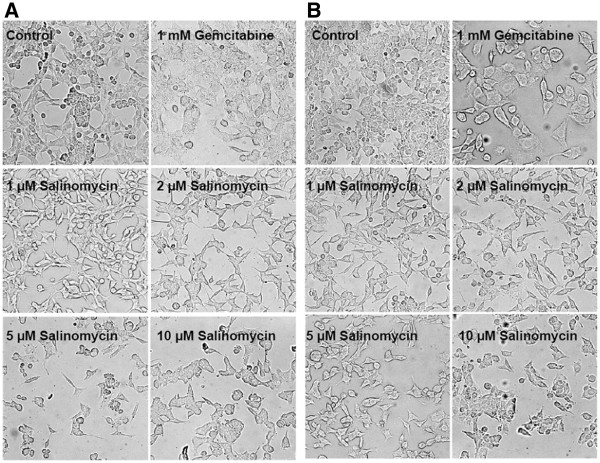
**Morphological changes after treatment with Salinomycin.** 3 x 10^4^ Mz-ChA-1 and EGI-1 cells were plated in chamber slides and further cultured until confluence. Cells were subsequently incubated in the absence or presence of 1 mM Gemcitabine, 1 μM, 2 μM, 5 μM or 10 μM Salinomycin for 24 hours. (**A**): Effects of Gemcitabine and Salinomycin treatment on Mz-ChA-1 cells. Incubation of cells with 1 mM Gemcitabine resulted in swollen and cobblestone-like appearance. In contrast the morphology of Mz-ChA-1 cells after treatment with low concentration of Salinomycin is altered to a haggard image and less confluent cell growth dependent on the amount of added agent. This appearance was further enhanced by higher concentration of 5 μM and 10 μM Salinomycin. (**B**): Treatment of EGI-1 cells with Salinomycin even with 5 μM and 10 μM revealed increased tolerance of agent since indication of morphological damages were less distinct. 1 mM Gemcitabine however caused comparable outcome on EGI-1 cells compared to Mz-ChA-1 and TFK-1 cells. Results are shown as representative pictures of one out of 3 independent experiments.

The described human CC cell lines Mz-ChA-1, TFK-1 and EGI-1 were exposed to increasing concentrations of Salinomycin (1 μM, 2 μM, 5 μM and 10 μM) for 24 hours. Gemcitabine as a broadly used chemotherapeutic for the treatment of CC was used as an interventional control in all performed experiments. After treatment with Gemcitabine, Mz-ChA-1 cells appeared swollen and grown in a cobblestone-like pattern (Figure 1 A). The administration of Salinomycin also altered the morphological appearance of the cells. While low concentrations of Salinomycin of 1 μM and 2 μM resulted in haggard and less confluent grown cells, increasing concentrations of Salinomycin of 5 μM and 10 μM resulted in a globular and defragmented cellular phenotype (Figure 1A). According to the results of exposure of Mz-ChA-1 cells to Salinomycin, also TFK-1 cells reacted in a similar pattern (data not shown). In contrast, exposure of Salinomycin to EGI-1 cells resulted in less characteristic morphological changes. While after treatment with 1 mM Gemcitabine, also swollen cells could be observed (Figure 1B), treatment of EGI-1 cells even with high concentrations of Salinomycin was accompanied by marginal morphological alterations and the lack of pronounced cell destruction. To figure out if the morphological changes of Mz-ChA-1 and TFk-1 cells are associated with an induction of apoptosis, we assessed the presence of apoptotic cells by Annexin V and TUNEL staining after treatment with Salinomycin.

### Salinomycin induces apoptosis in human CC cells

**Figure 2:**
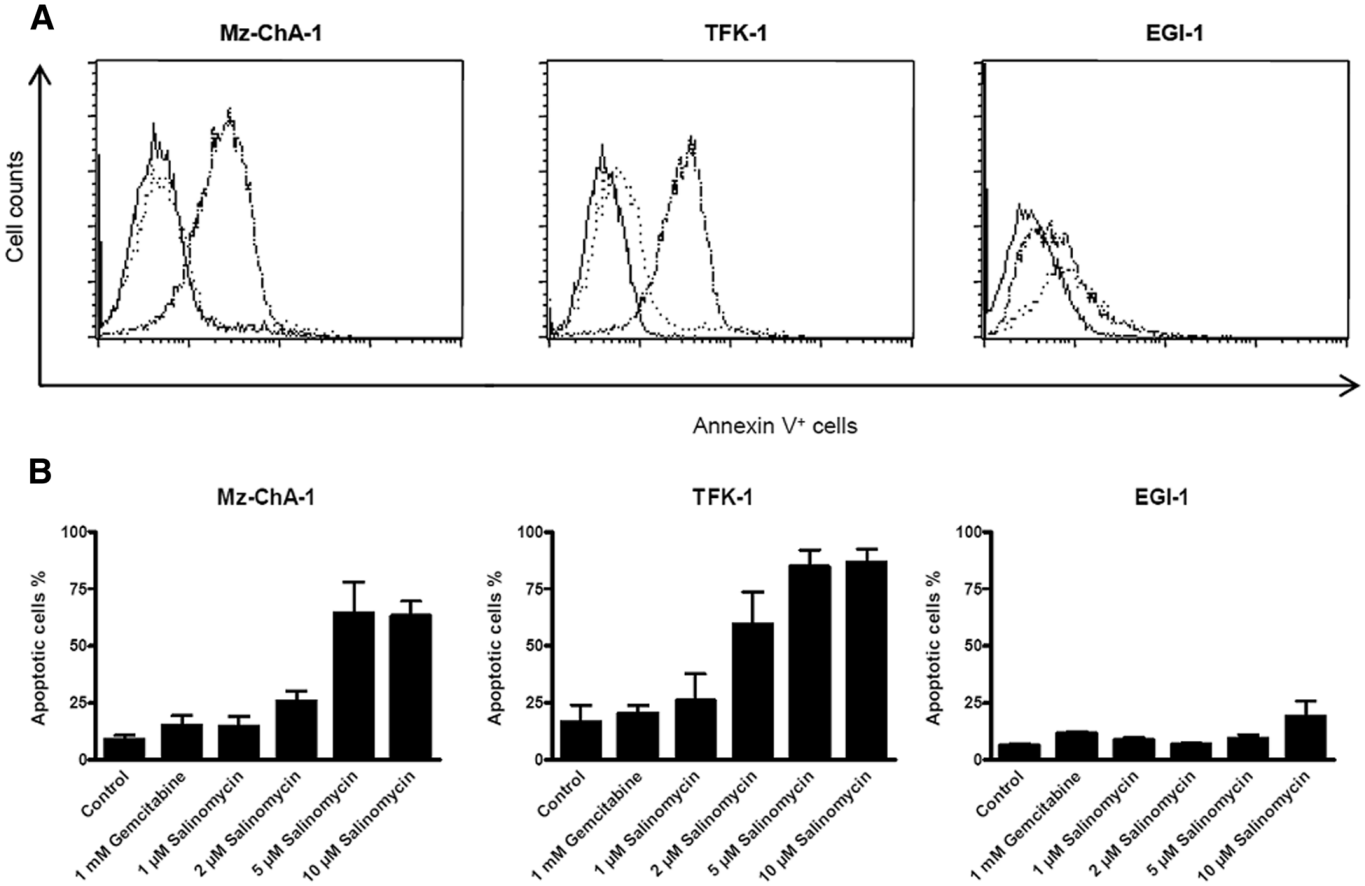
**Salinomycin induces apoptosis in human CC cells.** 1 x 10^6^ Mz-ChA-1, TFK-1 and EGI-1 cells were cultured until confluence, followed by subsequent incubation of cells in the absence or presence of 1 mM Gemcitabine, 1 μM, 2 μM, 5 μM or 10 μM Salinomycin for 24 hours. Cells were trypsinized and stained with Annexin V-FITC and analyzed by flow cytometry. (**A**) As expected, treatment with Gemcitabine (dotted line in overlay) led to very weak increase of apoptotic cells in all tested cell lines in comparison to untreated cells (solid bright line). In contrast, Salinomycin induced strongly augmented number of apoptotic cells for Mz-ChA-1 and TFK-1, whereas EGI-1 cells revealed a pronounced resistance towards Salinomycin-induced apoptosis (solid dark line). Results are shown as representative scatter-grams of Annexin V^+^ cells or summarizing 3 independent experiments as mean ± SD (**B**).

The rate of Annexin V positive human CC cells was determined by flow cytometry. Annexin V positivity is a typical attribute of apoptotic cells, thus an appropriate method to quantify apoptosis. As demonstrated in Figure 2, increasing concentrations of Salinomycin resulted in an increased percentage of Annexin V-positive Mz-ChA-1 and TFK-1 cells up to 65% and 85%, respectively (see Figure 2). Treatment with Gemcitabine resulted only in a low proportion of apoptotic cells. Interestingly, the ability of Salinomycin to induce apoptosis also in EGI-1 cells was noticeable reduced (Figure 2). These data represent the differences in the morphological appearance between Mz-ChA-1 and EGI-1 cells.

**Figure 3:**
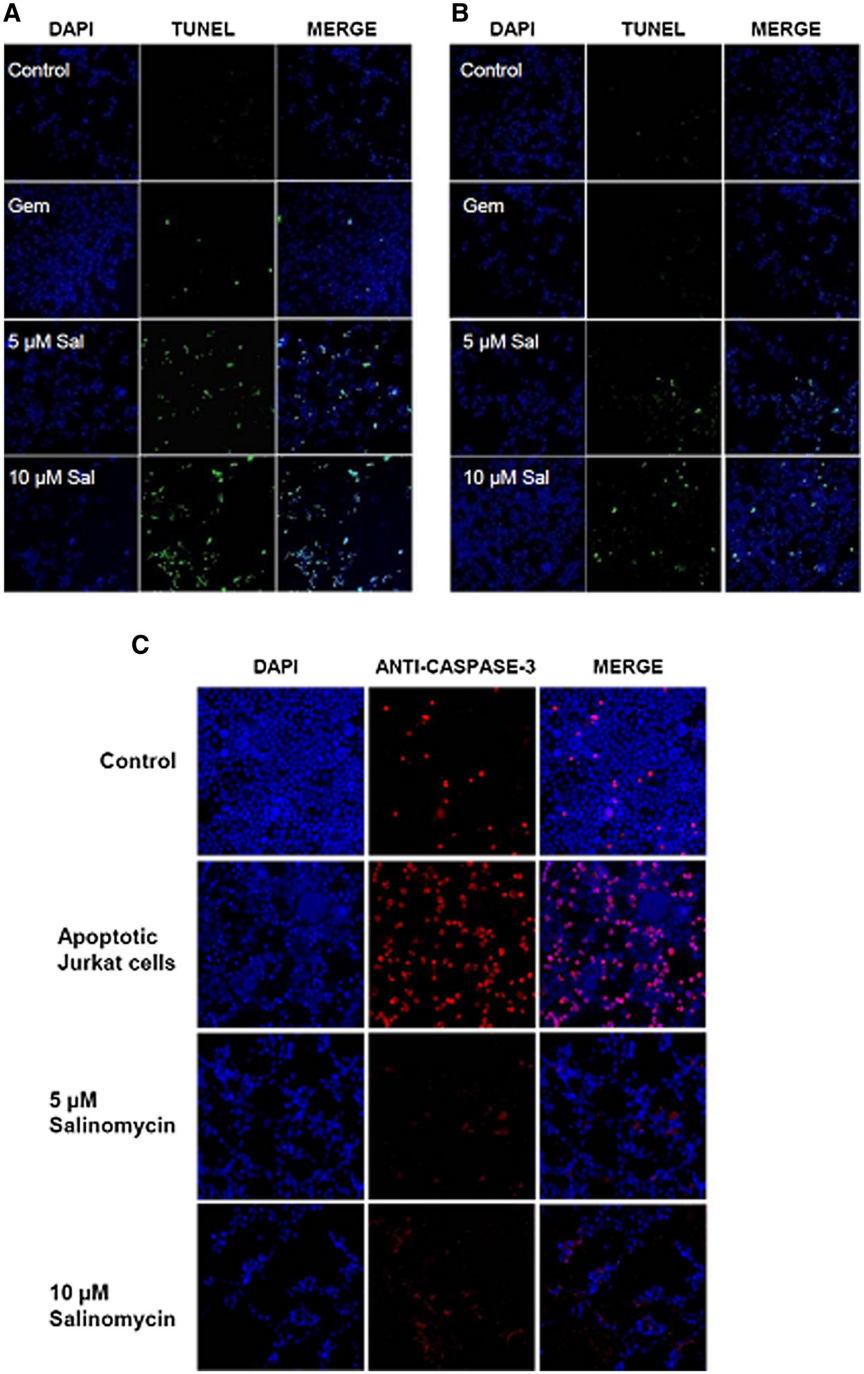
**Salinomycin induces apoptosis in human CC cells which is independent of activated caspases.** 3 x 10^4^ Mz-ChA-1 and EGI-1 cells were plated in chamber slides, cultured until confluence and treated as described before. (**A**) TUNEL assay of Mz-ChA-1 cells confirmed induction of apoptosis after exposure to increasing concentrations of Salinomycin whereas Gemcitabine failed to drive the cells into apotosis. (**B**) Assessment of TUNEL-positive EGI-1 cells revealed the drastic reduced number of apoptotic cells for this cell line. (**C**) Analysis of expression of Caspase-3 in Gemcitabine and Salinomycin treated Mz-ChA-1 cells. Although a distinct number of Mz-ChA-1 cells show apoptosis after Salinomycin treatment, Caspase-3 was barely traceable in treated cells. To proof quality of reagents and methods we used Jurkat cells that were exposed to TRAIL-expressing cells which induce reliable high percentages of apoptosis in Jurkat cells. Consequently a pronounced number of Jurkat cells were Caspase-3 positive. Results are shown as representative pictures of one out of 3 independent experiments.

To delineate the different outcomes of Salinomycin treatment on MZ-ChA-1 and TFK-1 cells on one side and EGI-1 cells on the other side, additional experiments were performed to assess apoptotic cells by TUNEL test. According to the detection of Annexin V-positive cells after treatment with Salinomycin for 24 hours by flow cytometry, high concentrations of 5 μM and 10 μM Salinomycin led to increased number of TUNEL-positive Mz-ChA-1 cells (Figure 3A). Elevated levels of TUNEL-positive cells were not detected in Mz-ChA-1 cells treated with Gemcitabine (Figure 3A). Similar findings were observed when TFK-1 cells were investigated (data not shown). In turn, TUNEL-positive staining in EGI-1 cells was less impressive compared to Mz-ChA-1 and TFK-1 cells (Figure 3B). These data are consistent with our previous finding of less Annexin V-positive EGI-1 cells.

Regarding that apoptosis is typically characterized by an activation of caspases we investigated if induction of apoptosis by Salinomycin in human CC cells is also accompanied by activated caspases. Therefore, we used a monoclonal antibody against caspase-3. Interestingly, activated caspase-3 was not found in Salinomycin-induced apoptosis in human CC cells (Figure 3C). Neither incubation of Mz-ChA-1 cells with 5 μM nor with 10 μM Salinomycin resulted in an activation of caspase-3. Also exposure of TFK-1 and EGI-1 cells to equivalent amounts of Salinomycin did not result in activated caspase-3. A non-effectiveness of the staining procedure was excluded by evidence of activated caspase-3 in apoptotic Jurkat cells after exposure to human TRAIL expressing transgenic fibroblasts (Figure 3C).

### Impaired tumor cell migration after treatment with Salinomycin

**Figure 4:**
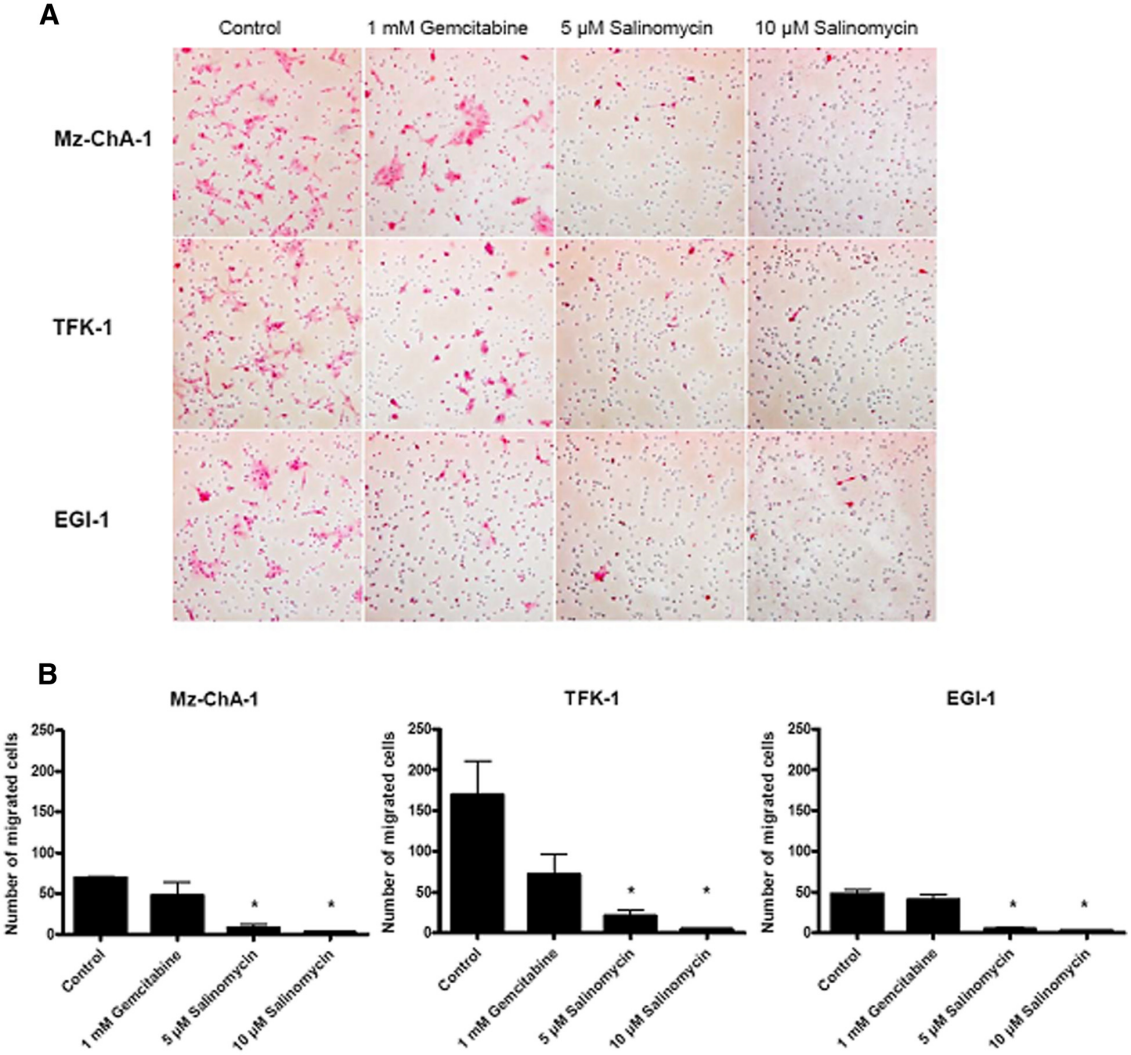
**Impaired migration after treatment with Salinomycin.** Tumor cell migration was analyzed using transwell chambers provided with an 8 μm pore polycarbonate membrane. Mz-ChA-1, TFK-1 and EGI-1 cells were placed at 5 x 10^5^ cells/well in culture medium containing 10% fetal calf serum in the upper compartment of the chamber. As chemoattractant, the lower compartment was filled with culture medium containing 30% fetal calf serum. Cells were cultured in the absence or presence of 1 mM Gemcitabine, 1 μM, 2 μM, 5 μM or 10 μM Salinomycin for 48 hours. The membrane was then removed, fixed with ethanol and stained with hematoxylin. The membranes were analyzed under a light microscope counting the number of migrated cells to the lower surface of the membrane in five random fields. Treatment with Salinomycin resulted in impaired migration in all three tumor cell lines. Results are shown as representative pictures of light microscopic display (**A**) and as mean ± SD (**B**) of 3 independent experiments. * p < 0,05 compared with control.

By demonstrating that Salinomycin induces apoptosis in human CC cells, we went in further detail of impaired cell function of CC cells after exposure to Salinomycin. One provisory attitude of tumor cells is migration. To delineate the ability of tumor cells to migrate from their original localization and therewith to form metastases, we investigated the ability of tumor cell migration in human CC cells after treatment with Salinomycin in a transwell chamber model. Mz-ChA-1, TFK-1 and EGI-1 cells were placed in transwell chambers provided with an 8 μm pore polycarbonate membrane and incubated with Salinomycin and Gemcitabine for 48 hours. Exposure to high concentrations of Salinomycin of 5 μM and 10 μM resulted in impaired migration in all three cell lines (Figure 4). Notably, the amount of migrated cells differed between the three different cell lines. These results were statistically significant. Treatment with Gemcitabine also resulted in impaired tumor cell migration. However, this difference was not significant. Consequently, after demonstrating that treatment with Salinomycin impairs tumor cell migration, we used the ^3^H-thymidine incorporation assay to assess if tumor cell proliferation is also affected.

### Enduring reduced proliferation of human CC cells after treatment with Salinomycin

**Figure 5:**
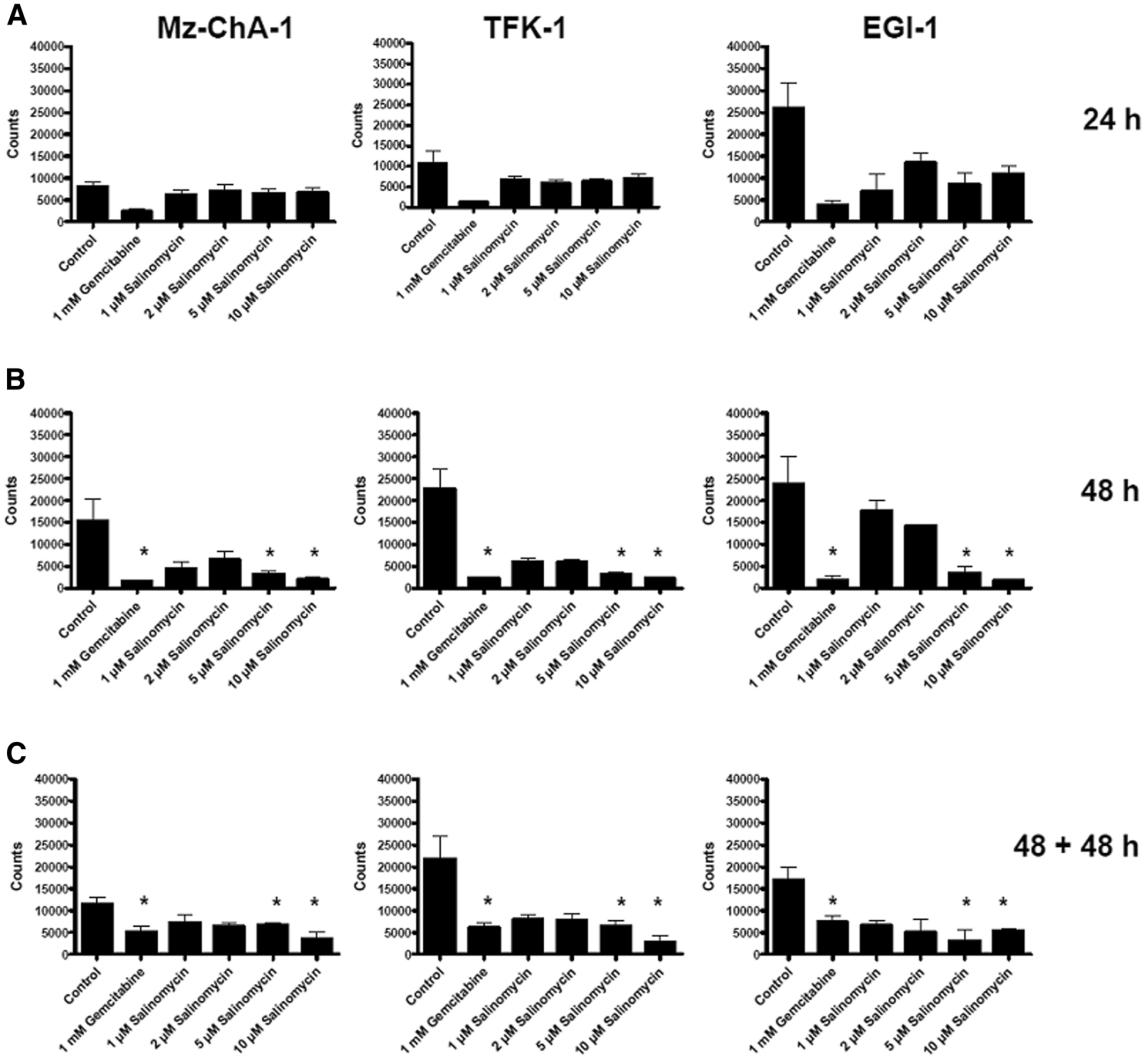
**Enduring reduced proliferation after treatment with Salinomycin.** 1 x 10^3^ human Mz-ChA-1, TFK-1 and EGI-1 cells were seeded in microtitre plates and grown until confluence. Cells were cultured in the absence or presence of 1 mM Gemcitabine, 1 μM, 2 μM, 5 μM or 10 μM Salinomycin. Treatment was performed for 24 (**A**) and 48 hours (**B**) or incubated for 48 hours under treatment and further grown with fresh medium for another 48 hours (**C**). After pulsing with ^3^H-thymidine, cell cultures were maintained for an additional incubation period of 16 hours. All three cell lines revealed significant reduced proliferation after 48 hours and high concentration of Salinomycin. This inhibition remained in treated cells even after removal of the agent. Results are shown as summary of 3 independent experiments as mean ± SD; * p < 0,05 compared with control.

Mz-ChA-1, TFK-1 and EGI-1 cells were treated with Salinomycin and Gemcitabine for 24 hours and 48 hours. Alternatively, cell culture was extended after exposure to the agents for 48 hours for an additional incubation period of 48 hours in medium alone. As demonstrated in Figure 5A, cell proliferation was impaired in all three cell lines after treatment with 1 mM Gemcitabine for 24 hours, due to the described anti-proliferative properties of Gemcitabine. Proliferation of Mz-ChA-1 and TFK-1 cells was barely impaired after exposure to Salinomycin for 24 hours. Interestingly, only proliferation of EGI-1 cells was reduced after exposure to high concentrations of Salinomycin of 5 μM and 10 μM. After treatment with 1 mM Gemcitabine for 48 hours, cell proliferation was significantly reduced in all three cell lines. Likewise, exposure to high concentrations of Salinomycin of 5 μM and 10 μM for 48 hours to Mz-ChA-1, TFK-1 and EGI-1 cells resulted in significantly reduced cell proliferation (see Figure 5B). To further evaluate the long-term effect of Salinomycin-administration to human CC cells, after incubation with Salinomycin for 48 hours, medium supplemented with Salinomycin was removed and the cells were further incubated for 48 hours in fresh medium alone. As demonstrated in Figure 5C, proliferation of human CC cells is sustainable reduced after incubation with high concentrations of Salinomycin, even 48 hours after lapse of the agent. These results were statistically significant. Although an ongoing resistance to apoptosis induction in EGI-1 cells could be observed after treatment with Salinomycin, we found strong indication of comparable properties of the agent to impair cell function of EGI-1 cells as observed in Mz-ChA-1 and TFK-1 cells. This raises the question of a potential impact of Salinomycin on the cell cycle of CC cells which in turn might explain the differences in outcome following treatment of the different cell lines.

### Salinomycin influences the cell cycle of human CC cells

**Figure 6:**
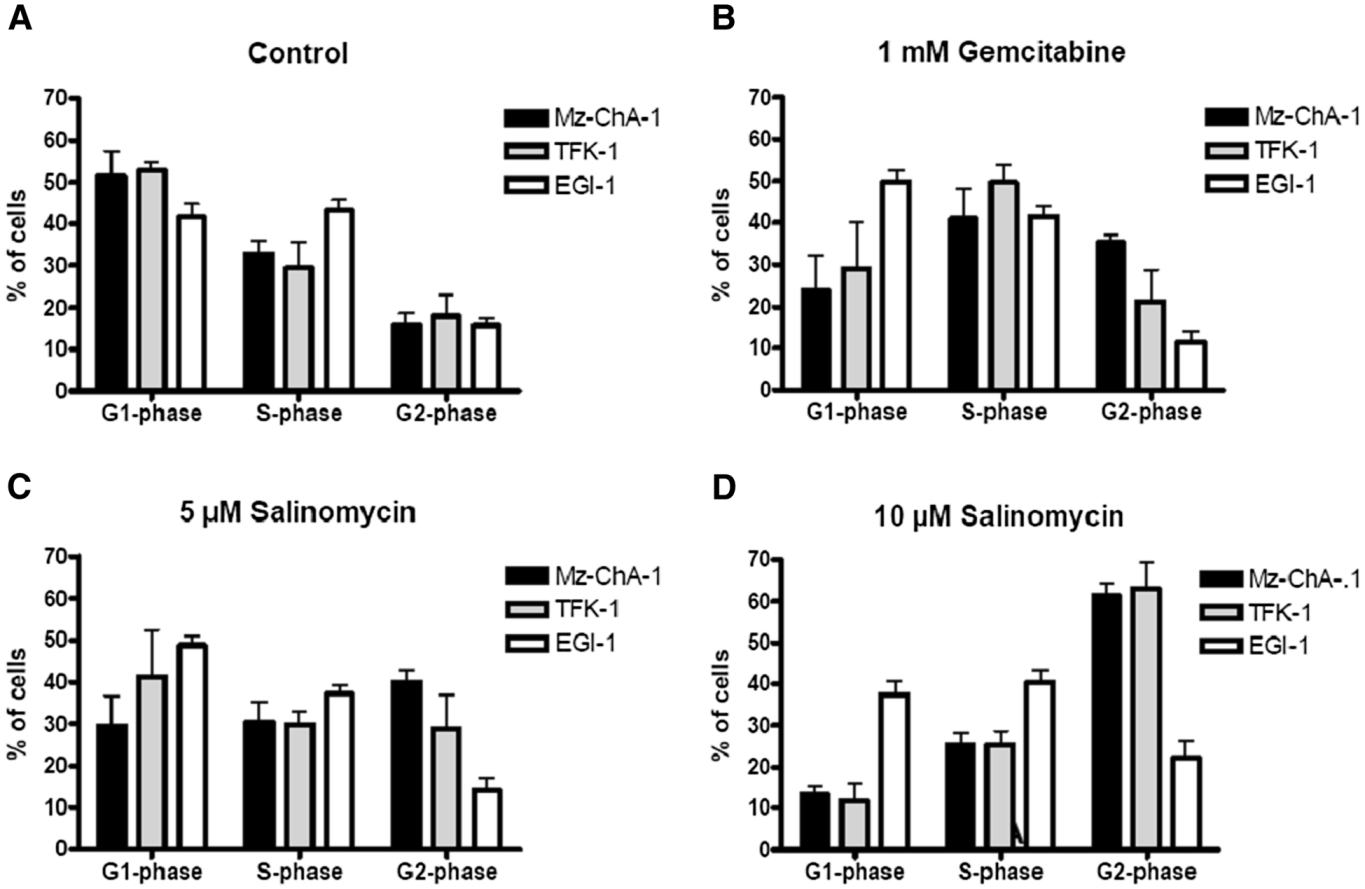
**Salinomycin causes accumulation of human CC cells in the G2-phase.** Mz-ChA-1, TFK-1 and EGI-1 cells were plated in 12-well plates with 1 x 10^5^ cells/well in culture medium. Cells were grown until confluence and then further cultured in medium (**A**) or presence of 1 mM Gemcitabine (**B**), 5 μM (**C**) and 10 μM Salinomycin for 24 hours (**D**). Cells were trypsinized and cell cycle was analyzed by flow cytometry. In Mz-ChA-1 and TFK-1 cells, increasing concentrations of Salinomycin resulted in accumulation of cells in the G2-phase. In contrast, differences in cell cycle phases were hardly observed in EGI-1 cells treated with Salinomycin. Results are shown as summary of 3 independent experiments as mean ± SD.

Mz-ChA-1, TFK-1 and EGI-1 cells were exposed to Salinomycin and to Gemcitabine for 24 hours. Cell cycle evaluation was based on propidium iodide staining and further analyzed using the ModFit LD software to assign the flow cytometric measurement to a defined cell cycle stage. As demonstrated in Figure 6, the cell cycle of untreated CC cells revealed a similar distribution for the growth of the three CC cell lines. Treatment with Gemcitabine resulted in an increased number of cells in the S-phase in regard of Mz-ChA-1 and TFK-1 cells while the cell cycle of EGI-1 cells did not alter in comparison to untreated cells. Interestingly, EGI-1 cells maintained the unaffected cell cycle even under treatment with high concentrations of 5 μM and 10 μM of Salinomycin apart from slight increase in the G1-phase after exposure to 5 μM Salinomycin. In contrast treatment of Mz-ChA-1 and TFK-1 cells resulted in drastic accumulation in the G2- phase (Figure 6A and B).

## Discussion

In this study we demonstrate that resistance to apoptosis of CC cells can be overcome by treatment with Salinomycin. We show that two of three cell lines respond to Salinomycin-treatment with a significant degree of apoptosis independent of Caspase-3 activity. In addition, Salinomycin inhibits cell proliferation and cell migration. Of note, this accounts for all three tested cell lines.

Patient´s survival suffering from CC is poor and even CC calls for up to 15% of all primary liver malignancies, the molecular pathogenesis is unclear to the greatest possible extent [2-4,26]. Consequently, characterization of the molecular pathogenesis and development of innovative therapeutic strategies are imperatively required particularly since current approaches such as administration of Gemcitabine combined with Cisplatin are rather part of a palliative concept than a curative treatment strategy [7]. This is most likely due to apoptosis resistance of CC cells and subsequently weak efficacy of common chemotherapeutical regimes. The induction of apoptosis in human CC cells is barely observed [25,27] or only detectable after co-treatment of the cells with additional drugs or inhibiting RNAs [28-30]. Accordingly, the understanding and the therapy of CC are characterized by nescience and ineffectiveness.

This is highlighted by the fact that even with Salinomycin which revealed capacity to provoke apoptosis in two of three tested human CC cell lines, EGI-1 remained to be unaffected in terms of being predispositioned to apoptosis. Exposure of Salinomycin to Mz-ChA-1 and TFK-1 cells, which were both originally isolated from an extrahepatic bile duct carcinoma [21,22], resulted in a high percentage of apoptotic tumor cells, while EGI-1 cells seem to be less susceptible for treatment with Salinomycin even after treatment with high concentrations. It has been reported that Salinomycin selectively affects malignant cells whereas non-malignant cells do not undergo apoptosis after treatment with Salinomycin [20,31,32]. Given that EGI-1 cells are originally isolated from a poorly differentiated human bile duct adenocarcinoma [23] and therewith undoubted are malignant, it remains unclear why these cells are nearly apoptosis-resistant to treatment with Salinomycin. These observations demand further investigations to elucidate potential escape mechanisms of tumor cells which might be important for a possible clinical application of Salinomycin in the future, indeed. Moreover, apoptosis-escape mechanisms of EGI-1 cells might explain in part the strong resistance of CC cells to chemotherapeutics in general.

However, the exact mechanisms by which Salinomycin induces apoptosis are still incomplete understood [15]. Salinomycin-induced apoptosis in human cancer cells is mediated by an uncommon pathway and independent of typical mechanism like activated caspases, death receptors like the CD95/DC95 ligand system or tumor suppressor protein p53 [15,19]. Demonstrating that Salinomycin-induced apoptosis in human CC cells is independent of caspase-3 activation confirms that apoptosis is mediated through an uncommon pathway. Given that caspase-3 is activated both in the extrinsic and intrinsic pathway of apoptosis and plays a predominant role [33,34], it is astonishing that none of the common pathways seems to be involved. Although activated caspase-3 can be found in apoptotic CC cells after treatment with Lobaplatin *in vitro*[35] another not yet discovered apoptotic pathway appears to be responsible for the effects of Salinomycin. Recently, it was reported that the Wingless type (Wnt)/β-catenin signaling pathway could be involved [31]. In chronic lymphocytic leukemia cells, Salinomycin inhibits the Wnt signaling cascade by blocking the phosphorylation of the Wnt co-receptor lipoprotein receptor related protein 6 (LRP6) causing impaired cell survival. These data are of great interest because in several tumor entities, LRP6 is over-expressed [36]. Even if not completely understood, Wnt signaling might also play an important role in the carcinogenesis of CC [37] and recently, the effectiveness of several Wnt pathway inhibitors on human CC cells has been demonstrated [38]. Additionally, it was reported that Salinomycin induces apoptosis in prostate cancer cells via accumulation of reactive oxygen species and mitochondrial membrane depolarization [39]. Furthermore, Salinomycin inhibits prostate cancer growth via reduction of the expression of key oncogenes and induction of oxidative stress in cultured prostate cancer cells [32]. Taken together, several mechanisms are supposed to be responsible for the effects of Salinomycin to human cancer cells, which have to be investigated in greater detail in the near future.

Furthermore, we demonstrate that the proportion of non-apoptotic tumor cells following Salinomycin-treatment is sustainable affected, characterized by impaired tumor cell migration, reduced proliferation and cell cycle accumulation. These observations are noteworthy due to well-known counterproductive reactions of tumor cells that escaped apoptosis, including hyperproliferation. To further characterize the effects induced by Salinomycin particularly on the continuous apoptosis-resisting EGI-1 cells, we investigated the ability of human CC cells to migrate after drug exposure. Tumor cell migration and therewith the ability to form metastases is a hallmark of tumors. While Ketola et al. have described impaired migration of prostate cancer cells after treatment with Salinomycin in a wound-healing assay [32]; this is the first report that migration through a membrane is effectively inhibited. These observations disclose an additional anti-cancer effect of Salinomycin in which all three cell lines are included.

Furthermore, the assessment of CC cell proliferation with or without Salinomycin treatment revealed a significant reduction of cell division in the presence of the agent. Again, all three cell lines, even EGI-1 cells, have shown this effect. We further demonstrate that Salinomycin- treatment of human CC cells induced an enduring reduced proliferation even after the abolition of treatment. This long-lasting effect demonstrates that the proportion of human CC cells that have escaped apoptosis after Salinomycin-treatment are affected permanently. These observations might be of particular importance for the potential clinical use of Salinomycin in the future as a prolonged effect of Salinomycin in patients with CC could also be expected.

In addition, we were able to correlate the anti-proliferative effects of Salinomycin with the results of the cell cycle analyses. The impact of Salinomycin on human CC cells is reflected by cell cycle accumulation in the G2-phase. This finding is noticeable because others have demonstrated that treatment with Salinomycin in equal concentrations is associated with accumulation in the pre-G1-phase, indicating increased apoptosis [17] Furthermore, in pre-treated human breast cancer with anti-mitotic drugs, Salinomycin abolishes G2-arrest and aneuploid cell formation [17,40]. In contrast, radiation-treated breast cancer cells accumulate in the G2-phase after treatment with Salinomycin [41]. Interestingly, Salinomycin-induced apoptosis in human leukemia cells is not accompanied by cell cycle arrest at all [20]. Thus, in respect to our data, it seems that the effects of Salinomycin on cell cycle are not consistent between human tumor cells of different origin. This again demonstrates the existing nebulosity concerning the biochemical mechanisms affected by Salinomycin.

Demonstrating the capability of Salinomycin to induce apoptosis and to interfere with tumor cell motility and proliferation in human CC cells, a potential and promising therapeutical approach for the treatment of CC might be discovered. Particularly, cancer entities with such calamitous prognosis like CC are tremendously dependent on innovative and sufficient therapy concepts. Thereby different human CC cell lines should be analyzed in regard to their susceptibility to Salinomycin-treatment. Furthermore, animal models have to be developed to investigate the impact of Salinomycin *in vivo*. To what extend Salinomycin will achieve to be a candidate for anti-cancer therapies in the future remains to be seen. Given that lethal intoxication in humans and animals are described [42-44], potential clinical studies must be planned very thoughtful. Thus, finding the dosage of Salinomycin will be crucial for its application in prospective therapeutical regimes.

## Conclusions

Salinomycin exhibits anti-tumor effects on human CC *in vitro*. Therefore, it should be considered as an innovative approach for the treatment of CC in the future and is worth to design further studies to proof practicability.

## Abbreviations

CC: Cholangiocarcinoma; HCC: Hepatocellular carcinoma; TUNEL: Terminal; dUTP: Desoxynucleotidy transferase nick end labeling; Wnt: Wingless type; LPR: Lipoprotein related protein.

## Competing interests

The authors declare that they have no competing interests.

## Authors’ contributions

TL and JK designed research; TL, WR, SB, and JK performed research, TL, JK, MW, and JK analyzed data; and TL and JK wrote the paper. All authors read and approved the final manuscript.

## Pre-publication history

The pre-publication history for this paper can be accessed here:

http://www.biomedcentral.com/1471-2407/12/466/prepub
